# Supplementary material for: Chitosan-Based Nanogels Designed for Betanin-Rich Beetroot Extract Transport: Physicochemical and Biological Aspects
Source: Polymers (Basel). 2023 Sep 25;15(19):3875. doi: 10.3390/polym15193875 (PMC10574865; doi:10.3390/polym15193875)
Supplement: Supplementary file 1 [file polymers-15-03875-s001.zip › polymers-2592610-supplementary.pdf]

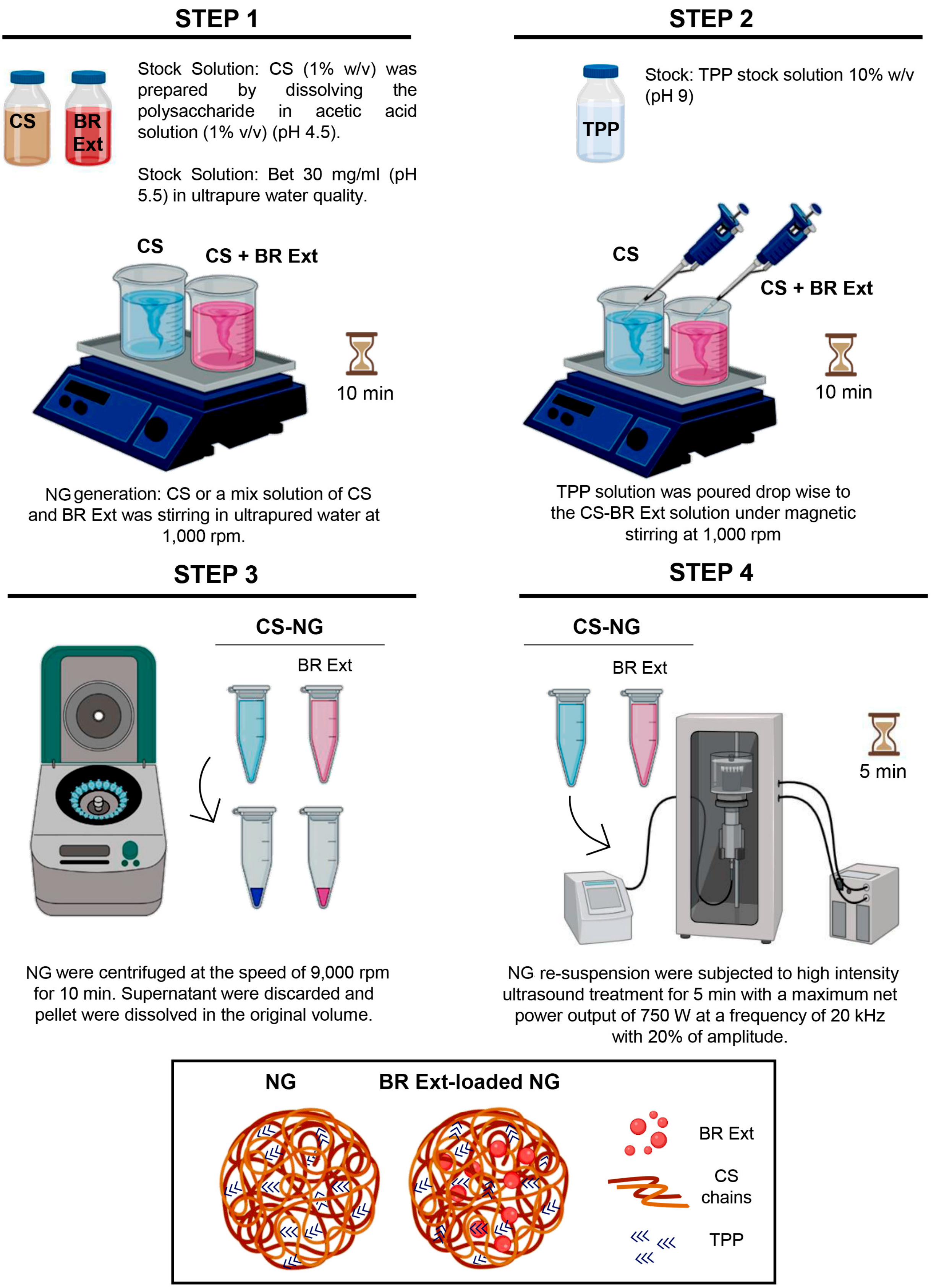

Figure S1. Diagram of NG generation protocol. Image created with BioRender.com.

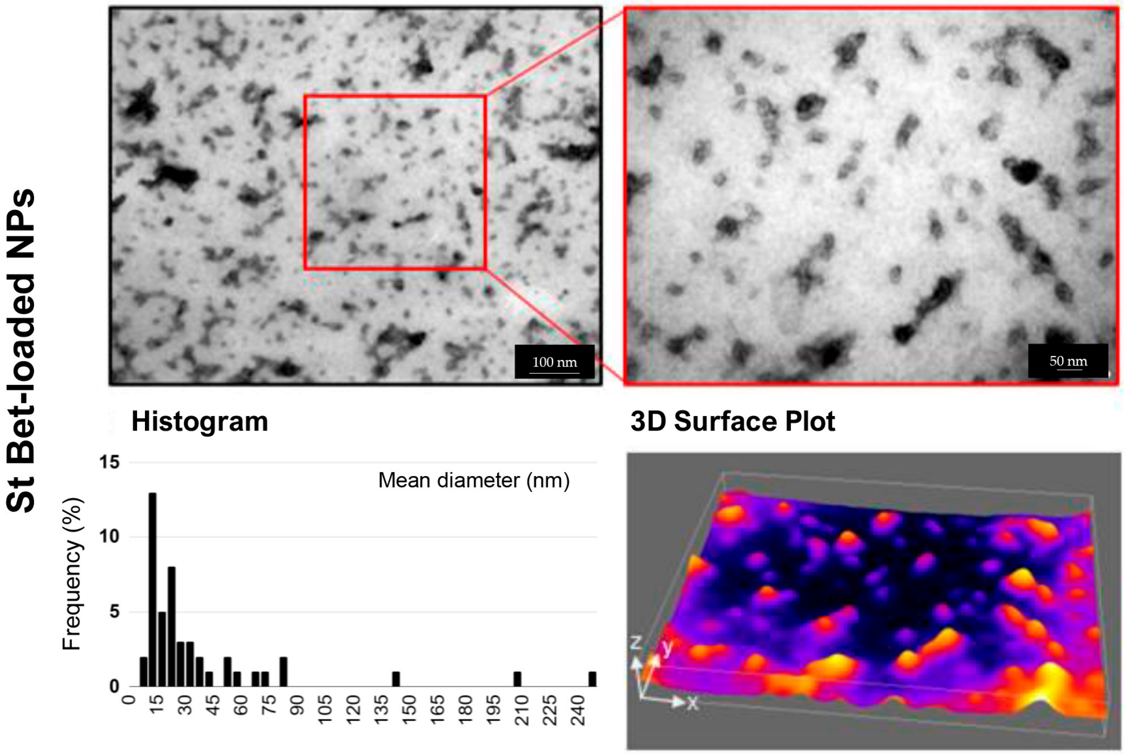

Figure S2. TEM images of St Bet-loaded NG sample with its frequency histograms and respective surface plots denoting the intensity of the NG in each case (Fiji 1.54f Software). Scale bar: 100 nm (30,000×); zoom: 50 nm (50,000×).
